# Racial Disparities in Premature Mortality and Unrealized Medicare Benefits Across US States

**DOI:** 10.1001/jamahealthforum.2025.4916

**Published:** 2025-11-07

**Authors:** Irene Papanicolas, Maecey Niksch, Jinru Wei, Reginald D. Williams, Jose F. Figueroa

**Affiliations:** 1 Department of Health Services, Policy and Practice, Brown School of Public Health, Providence, Rhode Island; 2 The Commonwealth Fund, New York, New York; 3 Department of Health Policy and Management, Harvard T. H. Chan School of Public Health, Boston, Massachusetts; 4 Department of Medicine, Brigham & Women’s Hospital, Harvard Medical School, Boston, Massachusetts

## Abstract

This cohort study examines racial disparities in premature mortality in the US and discusses the implications of these trends for unrealized Medicare benefits among populations with lower life expectancies.

## Introduction

Medicare is often regarded as a universal benefit for most US citizens and legal permanent residents. The program is financed through payroll tax contributions during individuals’ working lives with the expectation of coverage at age 65 years for most people.^1^ However, those who die prematurely are unable to realize the benefits they helped fund—raising equity concerns for populations with lower life expectancies.

In 2022, the mean life expectancy in the US was 77.5 years, down from 78.9 years in 2014,^2^ partly due to rising mortality among working-age adults and widening disparities across racial and socioeconomic groups.^3,4,5^ As health declines begin earlier^6^—especially for Black individuals—a growing number may not reach Medicare eligibility. This inequity in unrealized Medicare benefits underscores the need to understand how premature mortality before age 65 years has varied over time and by race.

## Methods

For this cohort study, using Centers for Diseases Control and Prevention (CDC) National Vital Statistics System microdata and CDC WONDER population estimates, we obtained state-level, all-cause deaths for adults aged 18 to 64 years in 2012 and 2022. We subtracted deaths of Medicare beneficiaries aged 18 to 64 years, identified from Medicare Beneficiary Summary Files, from total deaths among those aged 18 to 64 years in each state and calculated the premature mortality rate, age- and sex-standardized to the US population distribution (eMethods in Supplement 1). We examined overall premature mortality rates across US states and Washington, DC, for 2012 and 2022. Next, we compared premature mortality rates across states and by race (eTable in Supplement 1) for 2012 and 2022. Given differences in how race and ethnicity were coded across data sources, we restricted analyses to Black and White populations, for whom coding was most consistent and reliable. A 2-sided *P* < .05 was considered significant. Analyses were conducted in R, version 4.4.3 (R Foundation) and SAS, version 9.4 (SAS Institute). This study, conducted between March and May 2025, followed STROBE reporting guidelines and was approved by Brown University’s IRB, which waived informed consent given the use of deidentified data for a secondary analysis.

## Results

Premature mortality in the US increased from 243 to 309 deaths per 100 000 adults between 2012 and 2022, an increase of 27.2% (Table). Mississippi (2012) and West Virginia (2022) had the highest mortality rates, (337 and 488 deaths per 100 000, respectively), while Minnesota (2012) and Massachusetts (2022) had the lowest (185 and 225 deaths per 100 000, respectively). In analyses stratified by race, Black adults had higher premature mortality than White adults in 2012 (309 vs 247 per 100 000) and 2022 (427 vs 316 per 100 000). Age- and sex-standardized mortality rates increased by 118 deaths per 100 000 for Black adults (38.2%) and 68 deaths per 100 000 for White adults (27.7%) (Figure). Most states showed significantly higher premature mortality among Black vs White adults (all *P* < .05), except for New Mexico, Rhode Island, and Utah.

**Table.** Age- and Sex-Standardized Premature Mortality Across US States for All Races

State	Midyear population, adults aged 18-64 y of all races, millions		Standardized premature mortality (deaths per 100 000 adults aged 18-64 y, all races)		
	2012	2022	2012	2022	Difference
US total (population)^a^	197.03	203.04	243	309	66
Alabama	3.00	3.05	323	409	86
Alaska	0.48	0.46	288	398	110
Arizona	3.97	4.39	256	354	98
Arkansas	1.80	1.81	309	387	78
California	24.16	24.37	211	266	55
Colorado	3.35	3.71	219	287	68
Connecticut	2.26	2.23	196	254	58
Delaware	0.57	0.60	273	352	79
District of Columbia	0.46	0.46	315	375	60
Florida	11.79	13.15	258	307	49
Georgia	6.28	6.76	265	340	75
Hawai’i	0.88	0.85	226	259	33
Idaho	0.96	1.15	211	258	47
Illinois	8.12	7.70	237	302	65
Indiana	4.06	4.11	266	353	87
Iowa	1.88	1.89	219	272	53
Kansas	1.77	1.74	240	318	78
Kentucky	2.75	2.71	312	417	105
Louisiana	2.89	2.75	329	429	100
Maine	0.84	0.83	213	321	108
Maryland	3.78	3.77	242	298	56
Massachusetts	4.30	4.39	185	225	40
Michigan	6.18	6.04	251	309	58
Minnesota	3.37	3.43	185	244	59
Mississippi	1.84	1.75	337	450	113
Missouri	3.74	3.70	268	370	102
Montana	0.62	0.66	261	323	62
Nebraska	1.13	1.16	208	260	52
Nevada	1.73	1.95	282	342	60
New Hampshire	0.85	0.86	189	255	66
New Jersey	5.56	5.66	211	249	38
New Mexico	1.28	1.25	297	460	163
New York	12.54	12.12	206	251	45
North Carolina	6.13	6.54	249	343	94
North Dakota	0.44	0.47	227	318	91
Ohio	7.18	7.03	270	355	85
Oklahoma	2.34	2.41	333	414	81
Oregon	2.46	2.59	231	302	71
Pennsylvania	7.99	7.81	246	302	56
Rhode Island	0.68	0.68	200	237	37
South Carolina	2.95	3.16	294	386	92
South Dakota	0.51	0.53	234	330	96
Tennessee	4.05	4.29	309	420	111
Texas	16.25	18.55	251	300	49
Utah	1.69	2.04	214	230	16
Vermont	0.40	0.39	188	284	96
Virginia	5.26	5.35	221	287	66
Washington	4.41	4.83	207	274	67
West Virginia	1.16	1.05	327	488	161
Wisconsin	3.58	3.54	207	274	67
Wyoming	0.36	0.34	273	326	53

^a^ Population and mortality estimates are for the total US population aged 18 to 64 years. All rates in this table are rates at the population level; therefore, there are no margins of error to report.

**Figure.**
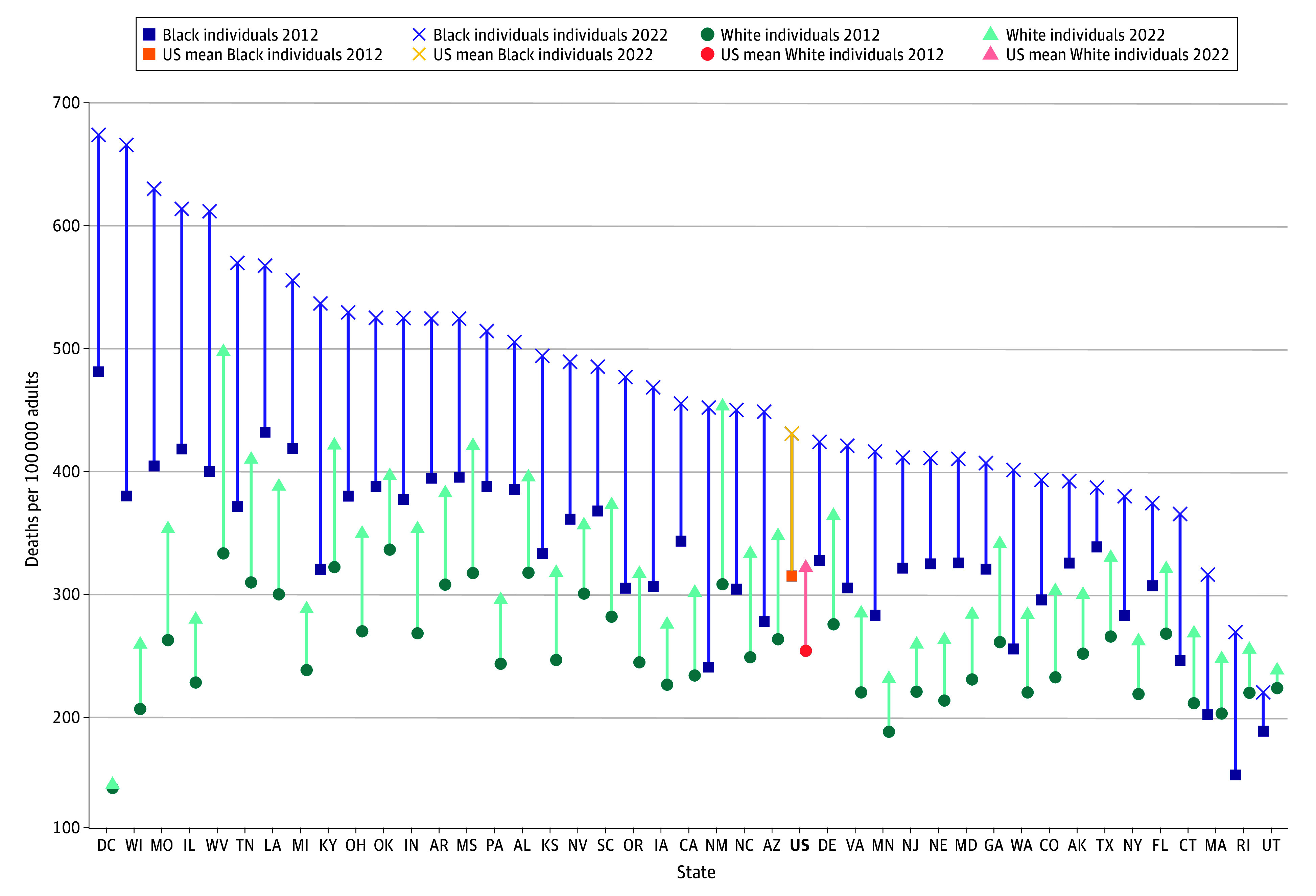
Age- and Sex-Standardized Premature Mortality Rates of Adults (Aged 18-64 Years) by Race Across US States, 2012 and 2022 Hawaiʻi, Idaho, Maine, Montana, New Hampshire, North Dakota, South Dakota, Vermont, and Wyoming were excluded due to data suppression requirements. All increases in premature mortality between 2012 and 2022 were statistically significant (*P* < .05) except for Black individuals in Texas, White individuals in Washington, DC, and Hawaiʻi, and for both racial groups in Utah.

## Discussion

Between 2012 and 2022, mortality among adults aged 18 to 65 years in the US increased by over 27%. During this period, racial disparities in premature mortality widened substantially, with Black individuals experiencing persistently higher and worsening rates compared with White individuals across most states. These results raise concerns about structural inequities within the Medicare entitlement and financing system. Despite contributing to Medicare throughout their working lives, Black individuals in the US are less likely to live long enough to reach the qualifying age for coverage.

This study’s limitations include analyses limited to Black and White individuals given known limitations and discrepancies in the coding of race and ethnicity between the CDC and Medicare data (eMethods in Supplement 1). This study highlights the need to confront structural inequities driving early mortality and to reconsider Medicare eligibility and health financing for populations with differing life expectancies. Reducing rising premature mortality and racial disparities, particularly among Black individuals, will require coordinated health and social policy reforms that ensure timely and equitable access to affordable health care coverage before 65 years of age, including access to Medicaid and private insurance, alongside sustained investments in factors that shape long-term health, such as housing, education, and income security.

## Notes

Supplement 1. **eMethods.** Elaboration on Data and Methodology **eTable. **Race Categories Across Datasets Used in Study
eReferences

Supplement 2.
Data Sharing Statement
