# Supplementary material for: Racial Disparities in Premature Mortality and Unrealized Medicare Benefits Across US States
Source: JAMA Health Forum. 2025 Nov 7;6(11):e254916. doi: 10.1001/jamahealthforum.2025.4916 (PMC12595528; doi:10.1001/jamahealthforum.2025.4916)
Supplement: Supplement 1. — eMethods. Elaboration on Data and Methodology eTable. Race Categories Across Datasets Used in Study eReferences [file jamahealthforum-e254916-s001.pdf]

Supplemental Online Content

Papanicolas I, Niksch M, Wei J, WIlliams RD, Figueroa JF. Racial disparities in premature mortality and unrealized Medicare benefits across US states. *JAMA Health Forum*. 2025;6(11):e254916. doi:10.1001/jamahealthforum.2025.4916

eMethods. Elaboration on Data and Methodology

eTable. Race Categories Across Datasets Used in Study

eReferences

This supplemental material has been provided by the authors to give readers additional information about their work.

© 2025 Papanicolas I et al. JAMA Health Forum.

eMethods. Elaboration on Data and Methodology

Data Sources

Mortality data for US states from 2012 and 2022 were obtained from CDC National Vital Statistics System (NVSS) Multiple Cause of Death restricted-use micro-data files.[1] Mid-year population estimates by state, single year of age, and race are from the CDC WONDER Bridged-Race Population Estimates[2] (2012) and Single-Race Population Estimates[3] (2022). CDC WONDER changes its race categorizations in 2021, which is why the estimates in 2022 came from the single-race population estimates. Estimates of the number of decedents aged 18 to 64 years who were Medicare beneficiaries were drawn from Master Beneficiary Summary File (MBSF) Base (2012 and 2022).

Estimating premature mortality

Using the mortality micro-data for US states, we tabulated the number of all-cause deaths by state (decedents' state of residence) across 15-year age groups (18-33, 34-49, and 50-64), sex, and race (White, Black, and "Other") in each year for 2012 and 2022. Since our interest was to determine premature mortality among people aged 18-64 years who were not enrolled in Medicare, we needed to account for and subtract any deaths of people ages 18-64 who were enrolled in Medicare across each state-age-sex-race category. More specifically, we tabulated the number of all-cause deaths by state, 15-year age group, sex, and race among Medicare beneficiaries aged 18-64 in each year for 2012 and 2022 using the Medicare Beneficiary Summary File data. We then estimated the number of decedents in each year—by state, 15-year age group, sex, and race—who died prematurely (prior to Medicare eligibility) by subtracting the number of Medicare beneficiary deaths from the number of total population deaths for people aged 18-64, also by state, 15-year age group, sex, and race.

Next, we calculated the crude premature mortality rate, dividing the number of decedents aged 18-64 who died prematurely with the mid-year population estimate for people aged 18-64 in each year by state, 15-year age group, sex, and race. Then, we multiplied these crude rates by the proportion of each 15-year age group and sex in the 18-64 overall US population in each year, adjusting the all-cause premature mortality rates for Black adults and White adults to the age-sex distribution of the 18-64 US population. We summed these adjusted rates across 15-year age group and sex within each racial group, state, and year to obtain the age-sex standardized, all-cause premature mortality rate by race in each state-year for adults aged 18-64, and we converted this to a rate per 100,000 people.

Similarly, we also calculated the premature mortality rates among all adults 18-64 years old in each state regardless of race, including adults from other race categories which we could not report individually. To do this, we calculated the crude premature mortality rate by dividing the number of decedents aged 18-64 who died prematurely with the mid-year population estimate of people aged 18-64 in each year by state, 15-year age group, and sex. Then, we multiplied these crude rates by the proportion of each 15-year age group and sex in the 18-64 overall US adult population in each year, adjusting all-cause premature mortality rates to the age-sex distribution of the 18-64 US population. We summed these adjusted rates to obtain the age-sex standardized, all-cause premature mortality rate for all races in each state-year for adults aged 18-64 and converted this to a rate per 100,000 people.

Analyses were conducted in R and RStudio (Version 4.4.3) and SAS 9.4. We followed STROBE Reporting Guidelines. This secondary data analysis was approved by the Brown University Institutional Review Board and determined exempt from review and informed consent.

Limitations in race and ethnicity classifications across data sources

There are inconsistencies between CDC NVSS and CMS data regarding the classification of race and ethnicity (**Table 1**). To limit the differences between these data, we focused on the two most common race categories of "Black" and "White." Prior research has shown that the Black race variable has a high validity, sensitivity, and specificity in administrative claims data.[4] For CDC NVSS data, any race other than "White" and "Black" alone, such as any of the multiple race categories, was categorized as "Other" in our

© 2025 Papanicolas I et al. JAMA Health Forum.

analyses (i.e., “Black and White” was not included in “White” or “Black”). Unfortunately, we were unable to report any descriptive findings for American Indian and Alaskan Native (AIAN) peoples, Asian-Pacific Islanders (API), Hispanics, or people with multiple racial identities, both due to differences in categorization across data sources and data suppression requirements for small cell sizes (0<n<10) per our Data Use Agreements (DUAs). We were also unable to report values for certain state-years by race due to data suppression requirements for the following states: Idaho, Hawai’i, Maine, Montana, New Hampshire, North Dakota, South Dakota, Vermont, and Wyoming. However, these states are included in for overall premature mortality rates calculated across all races.

© 2025 Papanicolas I et al. JAMA Health Forum.

eTable. Race Categories Across Datasets Used in Study

Data Source for Mortality	Years	Race Categories
CDC NVSS Mortality Micro-Data	2012 and 2022	White
		Black
		American Indian or Alaskan Native (AIAN)
		Asian Indian
		Chinese
		Filipino
		Japanese
		Korean
		Vietnamese
		Other or Multiple Asian
		Hawaiian
		Guamanian
		Samoan
		Other or Multiple Pacific Islander
		Black and White
		Black and AIAN
		Black and Asian
		Black and Native Hawaiian or Other Pacific Islander (NHOPI)
		AIAN and White
		AIAN and Asian
		AIAN and NHOPI
		NHOPI and White
		Black, AIAN, and White
		Black, AIAN and Asian
		Black, AIAN, and NHOPI
		Black, NHOPI, and White
		AIAN, Asian, and White
		AIAN, NHOPI, and White
		AIAN, Asian, and NHOPI
		Asian, NHOPI, and White
		Black, AIAN, Asian, and White
		Black, AIAN, Asian, and NHOPI
		Black, AIAN, NHOPI, and White
		Black, Asian, NHOPI, and White
		AIAN, Asian, NHOPI and White
		Black, AIAN, Asian, NHOPI, and White
CMS Medicare Beneficiary Summary File (MBSF)	2012 and 2022	Uknown
		White
		Black
		Other
		Asian
		Hispanic
		North American Native
**Data Source for Population Estimates**	**Years**	**Race Categories**
CDC WONDER Bridged-Race Population	2012	White
		Black or African American
		American Indian or Alaskan Native
		Asian or Pacific Islander
CDC WONDER Single-Race Population	2022	White
		Black or African American
		American Indian or Alaskan Native
		Asian
		Native Hawaiian or Other Pacific Islander
		More than one race

© 2025 Papanicolas I et al. JAMA Health Forum.

eReferences

1. National Center for Health Statistics (NCHS). CDC NVSS Multiple Cause of Death Mortality micro-data. Published online August 15, 2019. Accessed June 18, 2024. https://www.cdc.gov/nchs/nvss/dvs_data_release.htm

2. United States Department of Health and Human Services (US DHHS), Centers for Disease Control and Prevention (CDC), National Center for Health Statistics (NCHS). Bridged-Race Population Estimates, United States July 1st resident population by state, county, age, sex, bridged-race, and Hispanic origin, on CDC WONDER Online Database. Published online September 21, 2021. Accessed June 18, 2024. https://wonder.cdc.gov/wonder/help/bridged-race.html

3. US Census Bureau. Single-race Population Estimates, United States July 1st resident population by state, county, age, sex, single-race, and Hispanic origin, on CDC WONDER Online Database. Published online August 23, 2023. Accessed June 18, 2024. https://wonder.cdc.gov/wonder/help/single-race.html#

4. Jarrín OF, Nyandege AN, Grafova IB, Dong X, Lin H. Validity of race and ethnicity codes in Medicare administrative data compared to gold-standard self-reported race collected during routine home health care visits. *Med Care*. 2020;58(1):e1-e8. doi:10.1097/MLR.0000000000001216

© 2025 Papanicolas I et al. JAMA Health Forum.
