# Supplementary material for: Racial Disparities in Premature Mortality and Unrealized Medicare Benefits Across US States
Source: JAMA Health Forum. 2025 Nov 7;6(11):e254916. doi: 10.1001/jamahealthforum.2025.4916 (PMC12595528; doi:10.1001/jamahealthforum.2025.4916)
Supplement: Supplement 2. — Data Sharing Statement [file jamahealthforum-e254916-s002.pdf]

Data Sharing Statement

Papanicolas. Racial Disparities in Premature Mortality and Unrealized Medicare Benefits Across US States. *JAMA Health Forum*. Published November 07, 2025. doi:10.1001/jamahealthforum.2025.4916

Data
Data available: No

Additional Information
Explanation for why data not available: The data is not publicly accessible
